# Kidney disease in children and adolescents with perinatal HIV-1 infection

**DOI:** 10.7448/IAS.16.1.18596

**Published:** 2013-06-18

**Authors:** Rajendra Bhimma, Murli Udharam Purswani, Udai Kala

**Affiliations:** 1 Department of Paediatrics and Child Health, School of Clinical Medicine, Nelson R Mandela School of Medicine, University of KwaZulu-Natal, Durban, South Africa; 2 Division of Pediatric Infectious Disease, Albert Einstein College of Medicine, Bronx-Lebanon Hospital Center, Bronx, NY 10457, USA; 3 Chris Hani Baragwanath Hospital, University of Witwatersrand, Johannesburg, South Africa

**Keywords:** human immunodeficiency virus, kidney, children, adolescents, anti-retroviral drug toxicity

## Abstract

**Introduction:**

Involvement of the kidney in children and adolescents with perinatal (HIV-1) infection can occur at any stage during the child's life with diverse diagnoses, ranging from acute kidney injury, childhood urinary tract infections (UTIs), electrolyte imbalances and drug-induced nephrotoxicity, to diseases of the glomerulus. The latter include various immune-mediated chronic kidney diseases (CKD) and HIV-associated nephropathy (HIVAN).

**Discussion:**

The introduction of highly active anti-retroviral therapy (HAART) has dramatically reduced the incidence of HIVAN, once the commonest form of CKD in children of African descent living with HIV, and also altered its prognosis from eventual progression to end-stage kidney disease to one that is compatible with long-term survival. The impact of HAART on the outcome of other forms of kidney diseases seen in this population has not been as impressive. Increasingly important is nephrotoxicity secondary to the prolonged use of anti-retroviral agents, and the occurrence of co-morbid kidney disease unrelated to HIV infection or its treatment. Improved understanding of the molecular pathogenesis and genetics of kidney diseases associated with HIV will result in better screening, prevention and treatment efforts, as HIV specialists and nephrologists coordinate clinical care of these patients. Both haemodialysis (HD) and peritoneal dialysis (PD) are effective as renal replacement therapy in HIV-infected patients with end-stage kidney disease, with PD being preferred in resource-limited settings. Kidney transplantation, once contraindicated in this population, has now become the most effective renal replacement therapy, provided rigorous criteria are met. Given the attendant morbidity and mortality in HIV-infected children and adolescents with kidney disease, routine screening for kidney disease is recommended where resources permit.

**Conclusions:**

This review focuses on the pathogenesis and genetics, clinical presentation and management of kidney disease in children and adolescents with perinatal HIV-1 infection.

## Introduction

It is estimated that 3.4 million children were living with (HIV) infection at the end of 2011, 91% of whom are in sub-Saharan Africa [1]. This region accounts for more than 70% of global HIV infections although it has only 10% of the world's population, and thus bears an inordinate burden of this disease [2]. The widespread use of highly active anti-retroviral therapy (HAART), introduced in 1996, has dramatically decreased the incidence of HIV-associated nephropathy (HIVAN) although a clear benefit in non-HIVAN kidney disease has not been demonstrated [3–5]. In spite of this demonstrated effectiveness, only 28% of children in need of HAART worldwide actually receive it. With the long-term use of HAART, drug toxicity, advancing age and chronic viral infections has resulted in an increase in the overall frequency of kidney diseases in HIV-infected individuals [6,7]. Complications such as end-stage liver, kidney and heart disease are taking on prominent roles in the management of HIV-infected adults [8,9]. Nonetheless, a general lack of surveillance and reporting of kidney diseases in HIV-infected children exists in most developing regions of the world where HIV is highly prevalent [10]. In a large United States cohort, it was estimated that kidney disease complicating HIV infection is now among the ten most common non-infectious conditions occurring in perinatally HIV-infected children and adolescents in the HAART era, with an incidence rate of 2.6 per 100 patient-years [11,12].

The spectrum of kidney disease that occurs with perinatal HIV infection in children encompasses chronic glomerular disorders, such as HIVAN and HIV immune complex kidney disease (HIVICK), the thrombotic microangiopathies (including atypical forms of haemolytic uraemic syndrome and thrombotic thrombocytopenic purpura), disorders of proximal tubular function and acute kidney injury [13]. Early reports of childhood HIVAN in African-American children were from Miami and New York [14,15], three years after the first description of this condition in adults [3]. The unique histological feature of HIVAN in children is classical focal segmental glomerulosclerosis (FSGS) with or without mesangial hyperplasia in combination with microcystic tubular dilatation and interstitial inflammation [16,17]. Mesangial proliferative lesions secondary to immune complex deposits may also be present in some HIV-infected children with HIVAN [16,17]. All other forms of kidney diseases associated with HIV infection in children are collectively referred to as HIV-related kidney diseases hereafter.

In this article, we review the pathogenesis, clinical presentation and management of HIVAN and other HIV-related kidney diseases, including complications of HAART therapy.

## Pathogenesis of HIVAN

### The role of HIV-1 infection of the kidney

HIV viral burden and immunosuppression are well-established risk factors for the development of HIVAN and the main reasons behind the decline in its incidence with HAART [18–21]. Consistent with this clinical evidence is the fact that infection of kidney epithelial cells by HIV-1 is now thought to result eventually in HIVAN, and that the kidney is also a reservoir for HIV-1 [22]. What is unclear is how the virus enters these epithelial cells since glomerular podocytes and renal tubular cells do not express CD4 or other co-receptors. Nevertheless, in vitro studies have demonstrated efficient transfer of HIV-1 viral nucleic acid from T-cells to renal tubular epithelial cells. It is also postulated that injured glomerular podocytes undergo proliferation and apoptosis, and that the remaining podocytes hypertrophy and leave bare segments of basement membrane that promotes the development of the sclerotic lesions that characterize HIVAN [22]. A report on the pathogenesis of childhood HIVAN by Ray *et al*. also discussed the role of productive mesangial cell infection by HIV-1 [23]. Three groups of investigators were able to demonstrate infection of cultured mesangial cells by the virus [24–26], while two others were unable to do so [27,28]. Studies have shown that the HIV *nef* gene is important in the development of the glomerular lesions of HIVAN, in particular the dedifferentiation and proliferation of podocytes, which are otherwise terminally differentiated [29–31]. The HIV *vpr* genes have been implicated in the development of tubular pathology in HIVAN, predominantly through the induction of apoptosis and cell cycle arrests [32–35], and the HIV *tat* gene has been shown to have a potential role in podocyte dedifferentiation [36].

### The role of FSGS without an accompanying collapsing glomerulopathy

Histopathological findings of HIVAN vary in children compared to adults. Although collapsing glomerulopathy is a hallmark of the disease in adults, the unique microscopic features of HIVAN in children are defined as the presence of classical FSGS with or without mesangial hyperplasia in combination with microcystic tubular dilatation and interstitial inflammation. Mesangial proliferative lesions secondary to immune complex deposits may also be present in some children [16,37]. The early paediatric literature describes HIVAN without a collapsing glomerulopathy always being present on biopsy [14,15,38]. In two recent paediatric studies [13,18], the percentage of children with biopsy-proven HIVAN that showed a collapsing glomerulopathy with FSGS was 14% and 32.5%. The findings on histology include classic FSGS and mesangial proliferative glomerulonephritis, both of which have been reported by Ray *et al*. to be consistent with a diagnosis of HIVAN in children [23,37]. The collapsing variant of FSGS is a clinically and pathologically distinct variant of FSGS. Indeed the clinical progression of the two is different, with a rapidly progressive course observed in the collapsing variant that is typically seen with adult HIVAN. In children with HIVAN, most without a collapsing glomerulopathy [39], the clinical course of disease is not as aggressive, with slower progression to eventual end-stage kidney disease [23].

### The role of host genetics

Several studies have identified a genetic basis explaining the increased risk for kidney disease and the occurrence of HIVAN almost exclusively in African-Americans, a population with a four-fold increased risk for end-stage kidney disease [40]. Interest initially centred around genetic variations at a locus near the *MYH9* gene on chromosome 22 [41,42]. Later, two independent sequence variants G1 and G2 in the *APOL*-1 gene adjacent to the MYH9 gene were found to be highly associated with FSGS and HIVAN, with odds ratios of 17 and 29, respectively [43,44]. These risk alleles are present only on African chromosomes. The combined risk frequency of G1 and G2 polymorphisms in the Yoruba tribe in west Africa is 62% and the frequency of either allele in persons of west African origin is 35%, largely operating in a recessive manner. Interestingly, the heterozygous state protects the individual against *Trypansoma brucei rhodesiense*, whilst the homozygous state confers an increased risk for kidney disease, similar to the protective effect of sickle cell trait against malaria, at the cost of sickle cell disease in the homozygous state [43]. The percentages of one- and two-risk alleles for *APOL*-1 in self-identified African-Americans in a cohort of children and adolescents with perinatal HIV-1 infection followed in the Pediatric HIV/AIDS Cohort Study are 43% and 13%, respectively [45]. The two identified *APOL1* risk alleles were noted to be in strong linkage disequilibrium with the *MYH9* risk haplotype, and association between *APOL1* and kidney disease remained significant after further adjustment for this and other combinations of the *MYH9* alleles. The high frequency of *APOL1* risk alleles in African populations do not provide an explanation for the biological mechanisms leading to an increased risk of FSGS associated with these variants [22].

## Pathogenesis of HIVICK

HIVICK is thought to arise either by the trapping or deposition of circulating immune complexes in the parenchyma, or by *in situ* immune complex formation, described in a detailed report on four patients by Kimmel *et al*. [46]. These immune complexes comprise various HIV core and envelope antigens including p24 and glycoprotein 41 and 160, respectively, bound to IgG or IgA antibodies that are part of the polyclonal immune response produced against these antigens in HIV-infected patients. Also included in this category are other immune-mediated diseases such as IgA nephropathy, and a membranous or membrano-proliferative glomerulonephritis that may or may not be associated with hepatitis B and C virus infections. A “lupus-like” glomerulonephritis, in which light immunofluorescence and electron microscopic features of lupus glomerulonephritis in the absence of clinical systemic lupus erythematosus, and without the serologic markers that accompany this disease, is also seen in HIV-infected adults and children [47]. Although HIVICK occurs in African-Americans, this entity is more likely to be seen in Caucasians [48], and is not associated with the single-nucleotide polymorphisms implicated in the pathogenesis of HIVAN in African-Americans. HIVICK is not uncommon, and was found in 33% of kidney biopsies in children in a US cohort [18], whereas in South Africa there is a regional bias with HIVICK reported in 7% of paediatric biopsies from Cape Town and a much higher incidence of 51% in Johannesburg [49]. The reasons for the differences in the histological spectrum of the disease from these two regions remain to be explored.

## Clinical presentation of HIVAN and other glomerular diseases in children and adolescents with perinatal HIV-1 infection

Kidney disease in children occurs at all stages of HIV infection. Anti-retroviral (ARV) agents, antibiotics such as aminoglycosides, antifungals (amphotericin B), antivirals (acyclovir), anti-tuberculosis drugs, anti-inflammatory drugs and combinations of all these contribute to kidney disease.

The spectrum of kidney disease seen in HIV-infected patients is shown in Table 1. Glomerular pathology in children and adults in different countries and populations vary tremendously. In blacks from Africa, America and Europe and Hispanic populations, FSGS with or without collapsing glomeruli and microcystic tubular dilatation are common [23]. In their Caucasian counterparts, mesangial hyperplasia and immune complex-type disease predominates [50]. HIVAN is still the commonest cause of kidney disease in HIV-1-infected children and adolescents in other parts of the world, as evidenced by recent reports from KwaZulu-Natal in South Africa and Nigeria, with a higher incidence in males [13,51]. The exceptions were two studies in adults and children in which HIVICK was equal to or more prevalent [52,53]. Common to both is interstitial inflammation. However, in other adult studies from Cape Town, South Africa [54,55], and paediatric studies from South Africa and other regions of the globe, FSGS was the commonest histological type [13,15,56–58]. The incidence and natural history of HIVAN has been dramatically altered by HAART [21,59,60]. Once the commonest cause of kidney disease in adults and children, it is likely surpassed now by renal toxicity arising from ARV treatment in the United States.

**Table 1:** Spectrum of kidney disease in HIV-infected patients

Glomerular diseases
HIV-associated nephropathy-collapsing focal segmental glomerulosclerosis
Non-collapsing focal segmental glomerulosclerosis
Membranoproliferative glomerulonephritis (hepatitis C and cryoglobulinaemia)
Lupus-like glomerulonephritis
Membranous nephropathy (hepatitis B)
IgA nephropathy
Post-infectious glomerulonephritis
Diabetic nephropathy
Minimal change nephropathy
Amyloidosis
Nephrosclerosis
Thrombotic microangiopathies
Fibrillary glomerulonephritis
Anti-neutrophil cytoplasmic antibody-associated vasculitis and anti-glomerular basement membrane disease (rare)
**Interstitial diseases**
Acute or chronic interstitial nephritis
Lymphoma
Acute tubular necrosis
Pyelonephritis
**Medication-related**
Crystal nephropathy: indinavir, nelfinavir, atazanavir, intravenous acyclovir, sulfadiazine
Proximal tubulopathy (Fanconi syndrome): tenofovir, lamivudine, abacavir, Didanosine
Distal tubulopathy: amphotericin

Reproduced with permission from Usama E, Ana MS, Stcey AR, Fernando CF. Treatment of HIV-associated nephropathies Nephron Clin Pract. 2011;118:c346–54. Copyright © 2011 Karger AG, Basel.

Below we describe the clinical presentation of the most common forms of HIV-related glomerular disease.

## Glomerular disease

Haematuria and proteinuria and subsequent development of nephrotic syndrome and chronic kidney disease (CKD) represent the commonest manifestations of HIV-related glomerular disease (Table 1) [13]. CKD in children with HIV infection usually has an insidious onset [49]. The strategy to minimize kidney damage is by screening urine for proteinuria and even microalbuminuria. The mean duration from the onset of proteinuria to developing end-stage kidney disease in children with HIVAN varied from 8 months to 3 years depending on the geographical area and associated AIDS-defining illnesses in untreated patients. Thus, prognosis prior to the introduction of HAART in patients with CKD was very poor [15,37,56,61–63]. The reported rate of CKD in HIV-infected patients on presentation varied from 5 to 40% [13,51,53].

### Haematuria

Microscopic haematuria, with or without proteinuria was the commonest presenting symptom of kidney disease in two African studies; 75% and 50% with or without proteinuria, thus noting its importance as a sign or symptom in patients with HIVAN and other HIV-related kidney diseases [13,64]. If there is persistence of microscopic haematuria with or without HAART, the patient needs to be evaluated further for the degree of kidney involvement and warrants a kidney ultrasound, serum electrolytes, creatinine measurement and urine microscopy [49]. Once urolithiasis has been excluded and there is no clear explanation for the haematuria, a kidney biopsy must be considered.

### Proteinuria

Persistent proteinuria (≥1+ on urinary dipstick testing) is significant. Urine samples must be sent to the laboratory for a urine protein/creatinine ratio (uPCR) and a ratio of ≥0.2 (measured as mg/dL protein divided by mg/dL creatinine) confirms underlying kidney disease, which can be used to monitor response to HAART as shown by Chaparro *et al*. [64]. In this study, the degree of proteinuria degree of proteinuria was proportional to loss of kidney function and mortality increased with nephritoc-range proteinuria [64]. Severe proteinuria is more prevalent in black African children [13,65,66]. It is also associated with a higher mortality rate, especially in the presence of collapsing glomerulopathy on kidney biopsy [13,51,53,67].

Thus, it is imperative to test for proteinuria in all HIV-infected patients and if persistent, to perform a kidney biopsy. This is borne out by an adult study in which even microalbuminuria correlated with renal parenchymal disease with a prevalence of 36% in HIV-infected black African patients [66]. There are no equivalent paediatric studies showing similar results. In a study from Enugu, Nigeria none of the 154 HIV-infected and 154 HIV-uninfected children screened for microalbuminuria were positive [68]. In another study of HIV-infected non-febrile children without any symptoms of renal disease at Chris Hani Baragwanath hospital situated in Johannesburg, South Africa, the prevalence of microalbuminuria was 25%, but unfortunately none of these patients had a kidney biopsy [69].

## HIV-associated nephropathy

HIVAN is the most aggressive kidney disease affecting up to 10% of HIV-infected patients and is the primary form of HIV nephropathy seen in adults [11,12]. The true prevalence of paediatric HIVAN is not known as kidney biopsies have not been performed regularly in all HIV-infected patients with proteinuria [13,15,37,53,56,61–63,70] and haematuria, especially persistent microscopic haematuria. The following criteria were used for the diagnosis of HIVAN in children:

1. Persistent proteinuria defined as an uPCR≥0.2 for 3 months or more, in the absence of acute infection especially in children of African descent.
2. Urine sediment with urine microcysts (shed epithelial cells).
3. Highly echogenic kidneys as detected by serial renal ultrasound performed 3 months apart.
4. Black race with a clinical history of nephrotic-range proteinuria with or without oedema or hypertension.

### Diagnosis of HIVAN

All HIV-infected children should be screened for proteinuria and microscopic haematuria annually or earlier if indicated. The initial investigations should include blood urea nitrogen, serum electrolytes and creatinine, and urine electrolytes to evaluate for tubulopathies [14]. An uPCR must be done to assess the severity of proteinuria and to determine if nephrotic-range. Urine microscopy is done to determine the presence of microcysts which are clusters of renal epithelial cells forming cyst-like structures [37,56]. Ultrasound examination of the kidneys should be performed to assess kidney size, echogenicity and to exclude any obstructive lesions. Unfortunately, currently available non-invasive diagnostic testing has limited sensitivity and specificity to distinguish HIVAN from other HIV-related kidney diseases. Therefore, kidney biopsy should be performed, if indicated, to confirm the presence of HIVAN, which is presently the only definitive way to diagnose HIVAN [37]. In most United States paediatric centres, children with perinatal HIV-1 infection and kidney disease were not biopsied either because they were felt to be too ill to undergo the procedure, or because of the perception that information gained from the biopsy would not make a significant contribution to the management of these children [15,38,61,62]. To date, HIV infection has not been associated with an increased risk of procedural complications from kidney biopsy [71].

## Clinical presentation of other HIV-related kidney diseases in children and adolescents with perinatal HIV-1 infection

We describe below the presentation of some of the more commonly seen non-HIVAN kidney diseases that may accompany HIV-1 infection in this population.

## Acute interstitial nephritis

Acute interstitial nephritis (AIN) results mainly from multiple drugs used in the treatment of HIV infections and its complications. It can occur as a result of HIV infection of the kidney itself, as in 28% of autopsy findings in HIV-infected patients with AIN an inciting agent was not recognized [72]. Known agents causing AIN include non-steroidal anti-inflammatory drugs (NSAIDS), rifampicin and trimethoprim-sulfamethoxazole combinations [73,74]. It has also been reported in patients taking indinavir or ritonavir [57,75,76]. These protease inhibitors (PIs) can also cause nephrolithiasis with flank pain and renal colic [77]. Sulfadiazine crystal formation with resultant tubular obstruction and possibly ureteral obstruction has been described in volume-depleted HIV-infected patients [78–80].

Children with AIN from any cause, including HIV-infection, may present with non-specific signs and symptoms of acute kidney injury. More than 30 acute kidney injury definitions exist in the literature and therefore data may not be consistent, but a standardized definition has been proposed by the Acute Dialysis Quality Initiative Group [81] termed the “RIFLE” criteria and this has been modified for use in children [82]. RIFLE (the acronym for Risk for renal dysfunction, Injury to the kidney, Failure of kidney function, Loss of kidney function, and end-stage kidney disease) aims to standardize the definition of acute kidney injury by stratifying patients based on changes in serum creatinine levels from baseline and/or an abrupt decrease in urine output. There may be sudden or insidious onset of nausea, vomiting, and/or malaise. Many patients are asymptomatic. A minority of patients may have proteinuria and gross haematuria may be found in 5% of patients [83].

Discontinuation of the potential causative agent is the mainstay of therapy. In severe cases where there is persistent renal dysfunction, immunosuppressive therapy has been employed. However, the optimal therapy of AIN is unknown, since there are no randomized controlled trials or large observational studies. These complications can be prevented or minimized with ample fluid intake.

## Electrolyte and acid-base disorders

Electrolyte disturbances of hyponatraemia/hypernatraemia, hypophosphataemia, hypocalcaemia and hypomagnesaemia are common [13,53]. Hyponatraemia is often seen in HIV-infected children with gastroenteritis [84–86]. The syndrome of inappropriate anti-diuretic hormone secretion (SIADH) develops mainly in hospitalized patients [86] usually due to intracranial and respiratory infections such as pulmonary tuberculosis (TB), *Pneumocystis jiroveci* pneumonia and toxoplasmosis. Hyponatraemia and hyperkalaemia can be caused by adrenal insufficiency due to mineralocorticoid deficiency or hyporeninemic hypoaldosteronism [87,88]. Hypokalaemia due to low body potassium from severe malnutrition and gastrointestinal losses is also commonly seen. This also occurs through renal tubular loss resulting from the use of drugs such as amphotericin B used for the treatment of severe fungal infections. Toxicity from anti-retroviral agents such as tenofovir can cause proximal tubular dysfunction and nephrogenic diabetes insipidus can manifest as glycosuria, hypophosphateemia, proteinuria, acidosis and acute kidney injury [89–92]. Therefore, the dosing of nephrotoxic drugs should be adjusted to the estimated glomerular filtration rate in patients with acute kidney injury or CKD [93,94].

Acid-base disturbances are common in children with HIV infection and are due mainly to severe sepsis and drugs [13,94]. Lactic acidosis may possibly be due to drug-induced mitochondrial dysfunction reported with zidovudine, diadanosine, lamivudine and stavudine and which could be present in a mild form in 5–25% of patients [64]. Non-anion gap metabolic acidosis can result from intestinal loss of bicarbonate from diarrhoea or renal losses from drug toxicity, most commonly amphotericin B [73].

## Urinary tract infections

There is a higher prevalence of urinary tract infections (UTIs) in HIV-infected patients [53,57] ranging from lower tract involvement to pyelonephritis. UTIs in these patients seem to be due more to malnutrition than from immunosuppression due to HIV infection [95]. To prevent kidney damage, it is important to diagnose and treat UTIs appropriately. In a group of 60 children with HIV and renal involvement studied in Johannesburg, South Africa, 23% had UTIs [49]. The investigation and treatment of UTIs in HIV-infected children is based on standard guidelines used for management of HIV-uninfected children with UTIs [95].

## Exclusion of associated infections

Pulmonary and disseminated TB should be excluded in HIVAN. Nourse *et al*. demonstrated in four of their HIV-infected children with proteinuria as well as granulomatous lesions on histology compatible with TB, that proteinuria resolved on anti-TB drugs alone prior to the introduction of HAART [67]. TB was also a predominant finding in an adult Indian autopsy study in patients with AIDS [96]. The prevalence of TB in a cohort of 60 children at the Chris Hani Baragwanath Hospital was 33% [53]. The impact of other viral infections such cytomegalovirus (CMV), hepatitis B and hepatitis C on HIVAN has not been fully explored. One study of renal pathology in HIV-infected adult patients described CMV infection of the kidney as a cause of acute renal failure [97]. Hepatitis B virus resistance to lamivudine has been noted in kidney transplant recipients on HAART regimens containing lamivudine [98]. In patients with hepatitis C virus co-infection, clearance of the virus with interferon-ribavirin therapy should be attempted early, especially prior to transplantation, as immunosuppression exacerbates hepatitis C infection in kidney allograft recipients making management of HIV and hepatitis C virus co-infection particularly difficult [99].

## Treatment of HIVAN

Once kidney involvement is detected by renal dysfunction, proteinuria and/or haematuria, HAART needs to be commenced as soon as possible in accordance with WHO guidelines [100]. If already on HAART, it may be that their HIV disease is not well controlled, as evidenced by CD4 depletion and/or a high viral load, both being risk factors for HIVAN. In such a situation, appropriate resistance testing can guide subsequent HAART regimens. Associated infections such as TB, if present, must be appropriately treated. HAART is commenced after exclusion of tuberculosis to avoid immune reconstitution inflammatory syndrome (IRIS); however, ARVs may be started before TB is excluded in sick children [5,101–103]. This possibly arrests the rapid progression of kidney disease.

NRTIs are excreted in the urine unchanged, and therefore decreased dosing is often required in CKD stage III and above [104]. The threshold for dose reduction varies for different NRTIs; most NRTIs generally require dose adjustment at creatinine clearance below 40–60mL/min/1.73m^2^. The dosing for zidovudine is only reduced at creatinine clearance <15mL/min/1.73m^2^ whereas the dosing for abacavir remains unchanged at any level of kidney function [94]. Due to this variability, it is challenging to prescribe fixed-dose combinations of NRTIs in patients with reduced kidney function. Most non-nucleoside reverse transcriptase inhibitors (NNRTIs), PIs, fusion inhibitors, integrase inhibitors, and the β-chemokine receptor (CCR5) antagonists do not require dose modification with CKD [105,106].

HAART itself can cause acute kidney injury and progressive nephropathy [107–110]. Some patients with normal kidney function at baseline still progress to CKD despite HAART [111]. Thus, patients on HAART who show progressive kidney disease or signs of acute kidney injury must undergo a kidney biopsy. This also applies to those in whom potentially nephrotoxic drugs are being used and whose kidney function fails to improve upon discontinuation of the drug [112].

Angiotensin-converting enzyme (ACE) inhibitors and angiotensin receptor blocking agents (ARB) can be used as an adjunct for decreasing proteinuria provided the patient does not have a depleted intravascular space, often due to severe gastroenteritis, fluid loss from tubulopathy and/or severe nephrotic syndrome [13,15,56]. The introduction of HAART and angiotensin II blockade reduces progression to end-stage kidney disease [94,113]. However, to date, no completed randomized controlled trials or quasi-randomized controlled trials have been done providing evidence for treatment of HIVAN using ACE inhibitors or ARBs as adjunctive to HAART, although in most centres this is presently the standard of care [102,114]. Diuretics need to be used with caution as these agents can exacerbate intravascular volume contraction, further worsening the decline in glomerular filtration.

Steroid therapy has, in the short term, been shown to improve kidney function and proteinuria in HIVAN and in children treated for lymphoid interstitial pneumonitis; long-term effects of steroids on HIVAN are unknown. In resource-limited countries with a high prevalence of TB this could potentially cause exacerbation of, and overwhelming infection with TB, particularly when compliance is always an issue [49]. Also, in the absence of HAART, corticosteroids have not been shown to prevent the progression of HIVAN in children [15,38,56]. Steroids are therefore not currently recommended for the routine management of HIVAN.

The effects of other immunosuppressive agents such as cyclophosphamide, cyclosporine, azathioprine, mycophenelate mofetil, tacrolimus, are not known, but some have been utilized in a selective manner in certain patients [62].

In summary, co-morbidities such as UTIs, hypertension and electrolyte and acid-base disorders need to be treated aggressively. Avoidance of nephrotoxic drugs and combinations of ARVs that cannot be adjusted according to the patient's estimated glomerular filtration rate will help prevent further kidney damage.

## Renal toxicity arising from ARV drugs

Infants with perinatal HIV-1 infection are started on combination ARV therapy as soon as the diagnosis is established and will remain on medications for the rest of their life, given the current state of our knowledge on the treatment of HIV. It is therefore critically important to understand the toxicity profile of these drugs in order to be able to use them effectively and safely. Unfortunately, there is a paucity of data on such toxicity in children. A comprehensive review by Jao and Wyatt has described kidney toxicity reported with all classes of ARVs, except for the integrase inhibitors and the CCR5 antagonists [106]. Most PIs have, on rare occasions, been associated with the development of urolithiasis. This toxicity is notably most commonly reported with the use of indinavir. Crystalluria occurs in 20%, and nephrolithiasis in 3% of patients on this PI. Indinavir has also been reported to cause sterile pyuria and interstitial nephritis, as well as haematuria, renal colic, papillary necrosis, acute kidney injury and CKD. Due to the frequency of crystalluria and haematuria and the lack of a convenient paediatric formulation, this drug is rarely used in children and adolescents. The dose of atazanavir is now established in the paediatric population [115] and, along with the combination of lopinavir/ritonavir (Kaletra^®^), is a frequently used PI in children. Although cases of nephrolithiasis and interstitial nephritis have been reported with its use, the incidence of such toxicity is very low [116,117]. Similarly, the NNRTIs are metabolized by the hepatic cytochrome P450 system and have minimal nephrotoxicity, with rare reports of minimal change disease and urolithiasis with the use of efavirenz, and acute hypersensitivity reactions with the use of nevirapine. Lamivudine, didanosine and abacavir are nucleoside reverse transcriptase inhibitors (NRTIs) for which there are rare reports of Fanconi syndrome, and for the latter two, nephrogenic diabetes insipidus [118–122].

## Tenofovir and renal toxicity

Tenofovir is one of the most widely used ARV agents in the United States. Until recently, it was used only in children ≥12 years, provided their body weight was≥35 kg. It is now available as a powder and a low-dose tablet, and in 2012, received approval for use in children ≥2 years of age. It causes proximal renal tubular toxicity [123] and has been investigated far more extensively than other ARVs in order to better understand its renal safety profile. With acute tubular injury, there is reduced glomerular filtration rate, presenting as acute kidney injury [124]. The initial presentation of chronic tubular toxicity is the appearance of proteinuria, with glycosuria, phosphaturia and uricosuria, resulting in a complete or partial Fanconi syndrome [125]. In adult randomized controlled clinical trials, nephrotoxicity was observed in 1–2% of individuals [126]. It has been argued, however, that this is an artificially low estimate due to rigorous screening prior to participation in such studies. Cohort studies may better reflect the true renal safety profile of tenofovir in clinical care. There are few such studies in HIV-infected children and adolescents, and these are described in Table 2. They have shown results ranging from 86% proteinuria to no evidence of impaired glomerular or tubular function. One prospective double-blind placebo-controlled study showed no toxicity, while a recent large prospective cohort study showed a 2.5-fold increased risk of proteinuria with use of tenofovir for >3 years [134]. Thus, findings have been inconsistent.

**Table 2:** Recent pediatric studies of tenofovir toxicity in children and adolescents with perinatal HIV-1 infection

Author, year, location	Study design and duration/follow-up	Renal outcome measures	Age (years)	*N*	Findings
Vigano, 2007 Italy [127]	Prospective, open-label tenofovir use without control group, 24 months	Serum creatinine, phosphate; proteinuria, glycosuria, urine protein, albumin and α-1 microglobulin creatinine ratios and maximal tubular phosphate reabsorption ratios; estimated GFR (glomerular filtration rate)	4.9–18	27	No evidence of impaired glomerular or tubular renal function in tenofovir-treated subjects
Andiman, 2009 USA [128]	Prospective cohort study; median follow-up of six years	Three sequentially abnormal renal laboratory values in at least one of three measures: urine protein, serum creatinine or estimated GFR	Median age of 6.3	2102	Two-fold increased risk of renal dysfunction with tenofovir-based regimen
Judd, 2010 UK/Ireland [129]	Nested case–control study; median follow-up of 18 months	≥ grade 2 hypophosphataemia or estimated GFR<60 mL/min/1.73 m^2^	2–18	456	4% hypophosphataemia which was associated with prolonged tenofovir use; only one case of estimated GFR<60 mL/min/1.73 m^2^
Soler-Palacin, 2011 Spain [130]	Cohort study, two-phase design, retrospective and prospective data collection after≥6 months of tenofovir treatment without control group, median duration of tenofovir use: 77 months	Proteinuria>4 mg/m^2^/h, urine osmolality <800 mOsm/kg after restricted fluid intake, fractional Na excretion>2, tubular phosphate reabsorption<90%; estimated GFR; renal ultrasound alterations	8–17	40	Decreased tubular phosphate reabsorption in 74%; proteinuria in 89%; urine osmolality alterations in 22%; significantly decreased serum phosphate and potassium concentrations, with negative correlation between serum phosphate and time on tenofovir
Vigano, 2011 Italy [131]	Prospective, open-label tenofovir use without control group, 60 months	Same markers as 2007 study	4.9–18	26	Once again, no evidence of impaired glomerular or tubular renal function
Della Negra, 2012 USA, Brazil, Panama [132]	Prospective double-blind and placebo controlled (tenofovir versus placebo), 48 weeks	Serum phosphate and creatinine; urine protein and glucose; estimated GFR	12–< 18	87 45 tenofovir, 42 placebo	No significant differences in renal function between tenofovir and placebo group; no graded serum creatinine observed
Pontrelli, 2012 Italy [133]	Prospective cohort study, two years	Serum creatinine, phosphate and potassium levels, estimated GFR	9–18	49	Renal function and serum phosphate decreased over time in all subjects; no significant association with use of tenofovir (±protease inhibitors) containing regimens
Purswani, 2013 USA [134]	Prospective cohort study, three years	Urine protein/creatinine ratio≥0.2; CKD as ≥2 sequential uPCR≥0.2 or estimated GFR <60 mL/min/1.73 m^2^	Mean age (±SD) 11.5±2.5	448	2.5-fold increased risk of proteinuria with duration of tenofovir use>three years

## Dialysis in children with end-stage kidney disease

In the pre-HAART era, dialysis was not offered to patients with HIV infection because of poor survival and concerns regarding high infection rates in these children [49]. Following the introduction of HAART, several studies have confirmed short-term survival rates in adults that are similar to non-HIV-infected patients, such as diabetics [5,107]. Predictors of poor outcome of patients on dialysis with HIV-infection include low CD4 counts, high viral load, HIVAN as the cause of end-stage kidney disease, absence of HAART and opportunistic infections.

Given the improved survival of these patients with HAART, renal replacement therapy was shown to be feasible. Currently, there is still no consensus on the modality of dialysis that is best for HIV-infected children and adults. Both peritoneal dialysis (PD) and haemodialysis (HD) are effective modes of renal replacement therapy in these patients, though there are various points of concern with both modalities. In the United States, HD is preferred over PD because of the added burden of PD for family members who are often managing their own disease as well as that of their child [37]. Presently, both PD and HD have been used in HIV-infected patients with end-stage kidney disease, and the mode of dialysis is not a determining factor in the survival of adult HIV-infected patients with end-stage kidney disease [135,136]. While those patients on PD have a 50% increased risk of peritonitis, patients on HD using tunnelled cuffed catheters have a five-fold higher risk of infection with gram negative bacteria and a seven-fold higher risk of infection with fungal species [137]. PD may also aggravate the malnutrition and hypoalbuminaemia in HIV patients with severe wasting syndrome. HD patients, on the other hand, have a higher risk of thrombosis [138,139]. In resource-limited settings, PD may be the modality of choice mainly due to cost implications and distance from centres able to provide HD.

There is little data on the outcome of children with end-stage kidney disease secondary to HIV on maintenance dialysis. In the early stages of the epidemic when HAART was not available, Ortiz *et al*. reported that once full-blown AIDS develops in an HIV-infected patient on HD, survival was significantly decreased [140]. Following the introduction of HAART, Tourret *et al*. reported that the survival of HIV-infected adult patients on HD was not statistically different from non-HIV patients on HD. In this study, the factors associated with mortality were a high viral load and a history of opportunistic infections [141]. Gordillo *et al*. reported on 12 HIV-infected children with end-stage kidney disease on maintenance HD compared to 32 non-HIV-infected children over a five-year period [142]. Their main findings were that body mass index and cardiovascular disease were associated with increased mortality in the HIV-infected children. A negative correlation of mortality in HIV-infected children to CD8 counts was consistent with studies in adult HIV populations [141]. Children who died also had lower CD4 counts and higher viral loads, although this did not show statistical significance given the small sample size. However, this was consistent with studies in adult HIV-infected patients [141]. Given the high mortality from cardiovascular deaths in this group of children, the authors, in a subsequent report, proposed that routine echocardiography be periodically performed in HIV-infected children on renal replacement therapy. This would enable detection of subclinical increased left ventricular mass index, or reduced shortening fraction, both of which may be early predictors of mortality [143]. To date, there are no reports on the outcome of HIV-infected children with end-stage kidney disease on maintenance PD.

## Transplantation in HIV-infected children with end-stage kidney disease

Prior to the introduction of HAART, the morbidity and mortality of HIV-infected patients was too high to justify using scarce resources in transplanting HIV-infected patients [98]. There were concerns that immunosuppression may exacerbate HIV replication in an already immunocompromised patient resulting in rapid progression of disease and increased mortality [144]. The ability to suppress HIV replication with HAART, as well as improved prophylaxis and treatment of opportunistic infections, encouraged the transplant community to reconsider this option in HIV-infected individuals.

Further impetus was provided by the serendipitous finding that many of the commonly used immunosuppressive agents were also effective against HIV. Cyclosporine inhibition of interleukin-2-dependent T-cell proliferation may suppress HIV replication [145,146]. Furthermore, by binding to cyclophyllin A, cyclosporin prevents the formation of HIV gag protein/cyclophyllin A complex necessary for nuclear transport of HIV DNA [147,148]. A prospective study showed more rapid immune reconstitution in HIV-infected patients treated with cyclosporine and HAART versus cyclosporine alone [149]. Mycophenolate mofetil (MMF) inhibits inosine monophosphate dehydrogenase, a rate-limiting enzyme in the synthesis of guanosine nucleotides, markedly decreasing intracellular nucleotides in lymphocytes and monocytes as these cells lack a salvage pathway for generating purines, and thereby preventing replication of these cells [150–152]. Hence, MMF can provide synergistic action with nucleotide analogues such as abacavir and didanosine, which are often integral components of HAART therapy [152,153]. Of potential concern is the in vitro antagonism with stavudine and zidovudine that may inhibit the action of MMF. However, this has not been demonstrated in vivo [144]. Sirolimus inhibits the mammalian target of the rapamycin (mTOR) pathway by directly binding the mTOR Complex1 (mTORC1) that down-regulates the CCR5 receptor, which is the T-cell co-receptor for the HIV virion [154].

Transplants performed in HIV-infected patients on HAART show one-year graft and patient survival rates comparable to HIV-uninfected patients, although acute rejections are seen more frequently in the former, at a rate double that seen in those that are uninfected [144]. It has been postulated that this may be the result of immune dysregulation, but could also represent incomplete immunosuppression due to changes in overall drug exposure. Higher acute rejection rates have been observed in patients of African descent [155–157].

The “Transplant Study for People with HIV” (www.HIVtransplant.com) has proposed selection criteria that continue to evolve as more experience accumulates in this group of transplant patients [158]. The inclusion criteria for selecting a suitable kidney transplant recipient with HIV-infection include, *inter alia*:

- Meeting the standard criteria for kidney transplantation.
- In children, the percentage of CD4+T-cell is better than an absolute CD4+T-cell count in defining an intact immune system, hence modification of criteria to include a T-cell percentage. For children 1–2 years of age, the T-cell percentage must be >30%, and in children 2–10 years of age it must be >20%.
- Undetectable viral load (<50 copies/mL) for more than 6 months.
- No change in the HAART regimen for at least 3 months prior to kidney transplantation.
- There must be compliance to treatment for at least 6 months and caregivers and/or recipients must demonstrate willingness and an ability to comply with the immunosuppression protocol, ARV therapy and prophylaxis for opportunistic infections.
- In the case of pulmonary coccidiodomycosis, the recipient must be disease-free for at least 5 years prior to kidney transplantation and in the case of neoplasms, for at least 2 years.
- Female candidates of child-bearing potential must have a negative serum human chorionic gonadotropin pregnancy test 14 days prior to transplantation. All candidates must practice barrier contraception.
- The ability to provide informed consent and, for children between 7 and 12 years, signed assent. In the case of minors between the ages of 13 and 18 years, the minor and parent(s) must both provide informed consent. These ages may vary according to the laws and Institutional Review Boards of various regions.

Exclusion criteria include, *inter alia*, the following:

- Advanced-cardio-pulmonary disease.
- Active uncontrolled malignancy with reduced life span;
- Significant infection which may flare or reactivate with immunosuppression, such as tuberculosis, aspergillosis and other fungal infections, severe bacterial infections and active human papilloma virus infection.
- Documented progressive multifocal leukoencephalopathy.
- Epstein-Barr virus and human herpes virus 8 associated lymphoproliferative disease.
- Documented poor compliance.
- Failure to obtain informed consent or where required, assent.

Pharmacokinetic interactions between immunosuppresants and HAART agents can be profound with the most notable drug interactions occurring between ARV agents and immunosuppressive agents that induce or inhibit the P-glycoprotein 1 flux transporters and the cytochrome P450 3A (CYP3A4)-metabolizing enzymes found in the gut and liver [98]. Interactions can lead to unexpected increases or decreases in drug plasma levels and result in organ rejection, toxic adverse reactions of drugs and possible exacerbation of HIV replication. Patients on PIs and cyclosporine require only about 20% of the immunosuppressant dose of the latter drug normally administered to renal transplant recipients without HIV [98]. Patients on a ritonavir-boosted PI regimen require even lower doses of calcineurin inhibitors than patients on other HAART regimens [159]. In patients on tacrolimus or sirolimus using PIs as part of HAART, not only is the dose of these immunosuppresive drugs markedly decreased, but the interval of dosing needs to be increased more than five-fold [98]. Azole antifungal and macrolide antibiotics also inhibit the CYP3A4 system, increasing immunosuppressant levels of calcineurin inhibitors and sirolimus [160]. Patients taking steroids usually need proton-pump inhibitors for gastric ulcer prophylaxis. Since proton pump inhibitors can reduce intestinal absorption of Atazanavir, this PI must always be used in conjunction with a boosting dose of ritonavir. Zidovudine as a component of HAART used in combination with MMF could lead to additive myelosuppressive effects [161].

Post-transplant prophylaxis used in HIV-infected kidney transplant recipients is the same as that in HIV-uninfected recipients [98]. These regimens include prophylaxis for CMV and fungal infections (including *Pneumocystis jiroveci*) in the early postoperative period. For those patients with acute rejection treated with lymphocyte-depleting agents, prophylaxis regimen should be resumed for 3–6 months after discontinuation of the anti-rejection treatment.

Although HIV-infected adults are at increased risk for cancers such as Kaposi's sarcoma and non-Hodgkin's lymphoma, the rates of these cancers have declined with the introduction of HAART [162,163]. In adults, however, hepatocellular carcinoma rates have increased and this is probably related to increased longevity of patients with HIV co-infected with hepatitis B or C [164]. There are no similar reports in children.

In summary, kidney transplantation in HIV-infected patients treated with HAART has shown excellent graft and patient survival rates at 3–5 years [156,157,165]. Most issues revolve around interactions between ARV agents and the immunosuppressive agents used to prevent rejection. Opportunistic infections in these patients do not seem to have considerably increased although these patients have higher rates of acute rejection. Hepatitis B and C co-infection in adults remain a major concern, both in terms of treatment options and long-term effects on progression of liver disease [98].

Based on the current evidence, exclusion of children with HIV-infection from receiving a kidney transplant can no longer be justified.

## Screening for kidney disease in children and adolescents with HIV-1 infection

The life expectancy of HIV-infected patients with kidney disease has greatly improved following the introduction of HAART [105]. Progression to end-stage kidney disease with its attendant complications still remains a significant co-morbidity. Thus, early detection of kidney disease would enable clinicians to intervene in a timely manner.

Routine screening for kidney disease is therefore recommended, where resources permit. The guidelines implemented by the New York State Department of Health AIDS Institute include measuring estimated glomerular filtration rate, blood urea nitrogen and urinalysis at baseline and every six months in HIV-infected patients. For those on a tenofovir-containing regimen, this needs to be performed at baseline, one month and thereafter at least every four months (www.hivguidelines.org) [166]. It is important that this be tailored to the resources and facilities available in different parts of the world and is consistent with local guidelines. Additional screening evaluations, urine microalbumin/creatinine ratios for example, may be indicated with additional risk factors such as concomitant diabetes mellitus. All HIV-infected patients, even if asymptomatic for kidney disease, should be educated on the importance of ARV therapy in preventing HIVAN and monitoring for other causes of kidney disease, including medication-related nephrotoxicity, hypertension and diabetes [54,167].

## Conclusions

The differential diagnosis of the kidney diseases that are associated with HIV has expanded well beyond HIVAN. It includes toxicity from ARV and other therapeutic agents, immune complex-mediated kidney disease, and other comorbid unrelated kidney diseases. Given the broad differential diagnosis and inadequate sensitivity and specificity of non-invasive diagnostic testing, kidney biopsy is the gold standard for the diagnosis of HIVAN. There is increasing evidence that HAART improves kidney function in HIVAN although a clear benefit in non-HIVAN kidney disease has not been demonstrated. Kidney transplantation is now a viable alternative to dialysis in HIV-infected patients with end-stage kidney disease. Expanding access to HAART and further insights into the pathogenesis of HIVAN will help curb the devastating projected epidemic of kidney diseases, especially in the developing world. However, the most lasting impact on the epidemiology of this disease remains the prevention of new HIV infections.
